# A High Diffusive Model for Nanomaterials

**DOI:** 10.1007/s11671-010-9783-y

**Published:** 2010-09-28

**Authors:** P Di Sia, V Dallacasa

**Affiliations:** 1 Department of Computer Science, Faculty of Science, Verona University, Strada Le Grazie 15, 37134, Verona, Italy; 2 Faculty of Computer Science, Free University of Bozen, Piazza Domenicani 3, 39100, Bozen-Bolzano, Italy; 3 Accademia Nazionale dei Lincei, via della Lungara 10, Rome, Italy

**Keywords:** Correlation Functions, Diffusion, Frequency-Dependent Complex Conductivity, Nanostructures, Semiconducting Oxides, Montecarlo Simulation

## Abstract

Considerable attention is today devoted to the engineering of films widely used in photocatalytic, solar energy converters, photochemical and photoelectrochemical cells, dye-sensitized solar cells (DSSCs), to optimize electronic time response following photogeneration. However, the precise nature of transport processes in these systems has remained unresolved. To investigate such aspects of carrier dynamics, we have suggested a model for the calculation of correlation functions, expressed as the Fourier transform of the frequency-dependent complex conductivity *σ*(*ω*). Results are presented for the velocity correlation functions, the mean square deviation of position and the diffusion coefficient in systems, like TiO_2_ and doped Si, of large interest in present devices. Fast diffusion occurs in short time intervals of the order of few collision times. Consequences for efficiency of this fast response are discussed in relation to nanostructured devices.

## 

One of the most important aspects of nanostructures concerns charge transport, which can be influenced by particle dimensions and assume different characteristics with respect to those of bulk. In particular, if the mean free path of charges due to scattering phenomena is larger than the particle dimensions, one has a mesoscopic system, in which the transport depends on dimensions and one might correct the transport bulk theories by considering this phenomenon. These problems occur also in a thin film, in which the smallest dimension can be less than the free displacement and therefore require variations to existing theoretical transport bulk models. This situation occurs particularly in connection with metal oxide, like transparency, hardness, etc. Therefore a rigorous knowledge of transport properties is to be acquired. To establish the applicability limit of a bulk model and to investigate the time response of systems at nanoscale we have performed a new approach based on correlation functions obtained by a Fourier transform of the frequency-dependent complex conductivity of the system [1]. With this method it is possible to calculate these functions using experimental data obtained by various films, like TiO_2_ and ZnO also in the form of nanowires, which have increasing interest for their technological, chemical and biomedical applications and which are engineered to reach the desired technical features. Also, the mesoporous films play a very important role, for their applications in devices for energy generation, photocatalytic processes in environment remediations and for the useful electronic propertiestechniques, in particular the Time-resolved THz spectroscopy (TRTS) [2,3]. Starting from the Drude–Lorentz model [4,5] we have obtained directly the correlation function of velocities, the quadratic average distance crossed by the charges as a function of time and the diffusion coefficient *D*.

From a mathematical viewpoint, the Kubo relation of the linear response must be inverted. But, due to the presence of a half Fourier-transform, it is necessary to modify this relation in such a way that the whole time axis (-∞, +∞) occurs. This procedure is not trivial and not previously found in the literature.

This new formula can be obtained by relying on linear response theory; we have started considering a system with an hamiltonian of the form:

(1) $$H=H_{0}+H_{1}$$

with *H*_1_ having small effects respect to *H*_0_, and negligible in the remote past (adiabatic representation). In the case of an electric field of frequency *ω* we have:

(2) $$H_{1}=e\vec{E}\cdot \vec{r}$$

For an electric field constant in space and depending on time as:

(3) $$\vec{E}={\vec{E}}_{0}{\text{e}}^{i\omega t}$$

the time dependent corresponding current is:

(4) $$\vec{J}\left(t\right)=\sigma \left(\omega \right)\vec{E}\left(t\right)$$

Following the standard time-dependent approach [4], we derived a general formula for the linear response of a dipole moment density $\vec{B}=e\vec{r}/V$ in the *β* direction with the electric field $\vec{E}$ directed in the *α* direction, where *V* is the volume of the system. This permits to deduce the susceptibility *χ*(*ω*), which is correlated to *σ*(*ω*) via the relation:

(5) $$1+4\pi \chi \left(\omega \right)=1+4\pi i\frac{\sigma \left(\omega \right)}{\omega }$$

From Eq. 5 we have deduced the real part of *σ*(*ω*), denoted in the form *σ'*(*ω*) as:

(6) $${\sigma^{\prime }}_{\beta \alpha }\left(\omega \right)=\frac{e^{2}\omega \pi }{V\hbar }S_{\beta \alpha }\left(\omega \right)\left(1-{\text{e}}^{-\hbar \omega /KT}\right)$$

where *S*_*βα*_(*ω*)is the quantity:

(7) $$S_{\beta \alpha }\left(\omega \right)=\int_{-\infty }^{+\infty }\text{d}t{\langle {\vec{r}}^{\alpha }\left(0\right){\vec{r}}^{\beta }\left(t\right)\rangle }_{T}{\text{e}}^{-i\omega t}$$

The quantity 〈···〉_*T*_ is the thermal average, and the exponential factor arises from equilibrium thermal weights for Fermi particles. By considering the identity $v=\frac{d}{\text{d}t}r=\frac{i}{\hbar }\left[H,r\right]$, Eq. 6 can be written in a form containing the velocity correlation function instead of the position correlation function. Assuming the high temperature limit *ħω* < <*KT* as usual in systems to be considered in this paper, we obtain:

(8) $${\sigma^{\prime }}_{\beta \alpha }\left(\omega \right)=\frac{e^{2}}{2VKT}\int_{-\infty }^{+\infty }\text{d}t{\langle {\vec{v}}^{\alpha }\left(0\right){\vec{v}}^{\beta }\left(t\right)\rangle }_{T}{\text{e}}^{-i\omega t}$$

The integral in Eq. 8 spans the entire *t* axis, so we can perform the complete inverse Fourier transform of this equation. It gives:

(9) $$<{\vec{v}}^{\alpha }\left(0\right){\vec{v}}^{\beta }\left(t\right){>}_{T}=\frac{KTV}{\pi e^{2}}\int_{-\infty }^{+\infty }d\omega \sigma_{\beta \alpha }^{\prime }\left(\omega \right){\text{e}}^{i\omega t}$$

with *V* the volume of the system, *K* the Boltzmann's constant, *T* the temperature and *σ'*(*ω*) the real part of *σ*(*ω*), given by:

(10) $$\sigma^{\prime }\left(\omega \right)=\frac{\tau ne^{2}}{m}\frac{\omega^{2}/\tau^{2}}{{\left(\omega_{0}^{2}-\omega^{2}\right)}^{2}+\omega^{2}/\tau^{2}}$$

where *n* is the carrier density, *ω*_0_ the proper oscillator frequency, 1/*τ* the collision frequency [4,5].

The mean squared displacement in relation with the correlation function of velocities is given by:

(11) $$\langle {\left[\vec{R}\left(t\right)-\vec{R}\left(0\right)\right]}^{2}\rangle =2\int_{0}^{t}\text{d}t^{\prime }\left(t-t^{\prime }\right)\langle \vec{v}\left(t^{\prime }\right)\cdot \vec{v}\left(0\right)\rangle$$

By integration of Eq. 9 with Eq. 10, we deduced all the results for $\langle \vec{v}\left(0\right)\cdot \vec{v}\left(t\right)\rangle$, *R*^2^(*t*) and $\frac{d}{\text{d}x}R^{2}\left(t\right)$ with *x* = *t*/*τ *[6].

The main advantage of this new formulation is the disposal of exact results for describing the dynamic behaviour, as extracted by time-resolved techniques. In our analytical procedure we have distinguished the case *ω*_*o*_ = 0 from the case *ω*_*o*_ ≠ 0. For this latter, three cases occur in connection with the sign of the quantity $\Delta =4\tau^{2}\omega_{0}^{2}-1$. After obtaining the respective *σ'*(*ω*), we have found the poles of these functions and then the residues for integration in the complex *ω*-plane via Cauchy theorem.

We have used our results for discussing transport in a conventional semiconductor such as doped Si, non conventional TiO_2_ and other systems where anomalous transport has been found.

The most important characteristics of the results are illustrated by concrete examples in Figures 1, 2, 3, 4, and 5.

**Figure 1:**
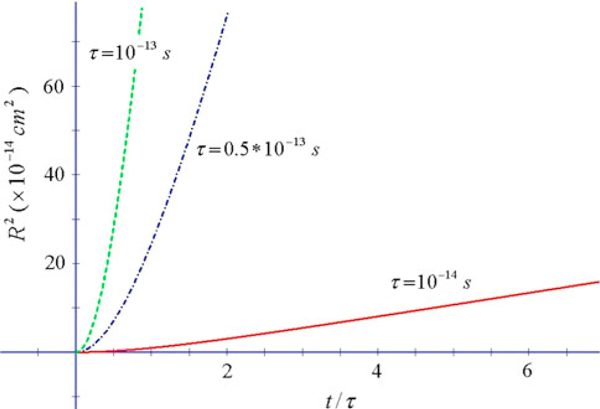
***R*^2^ vs. *x* = *t*/*τ* for some representative values of *τ*, typical of doped Si **[7]**(ω_0_ = 0, *T* = 300 K) (Drude model)**. A complete description of *R*^2^ for Si requires the evaluation of the contribution of the Drude–Lorentz part (see text).

**Figure 2:**
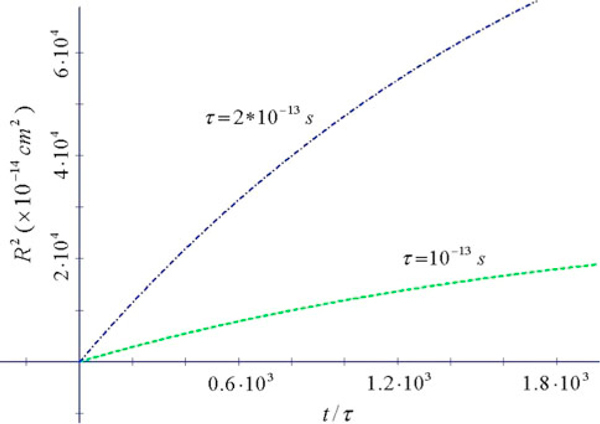
***R*^2^ vs. *x* = *t*/*τ* for 2 values of *τ* (*ω*_0_ = 1.12 × 10^11^Hz *dot-dashed*; *ω*_0_ = 2.24 × 10^11^Hz *dashed*) for TiO_2_ (*m* = 6*m*_e_, *T* = 300 K)**. Saturation values occur at sufficiently large *t*.

**Figure 3:**
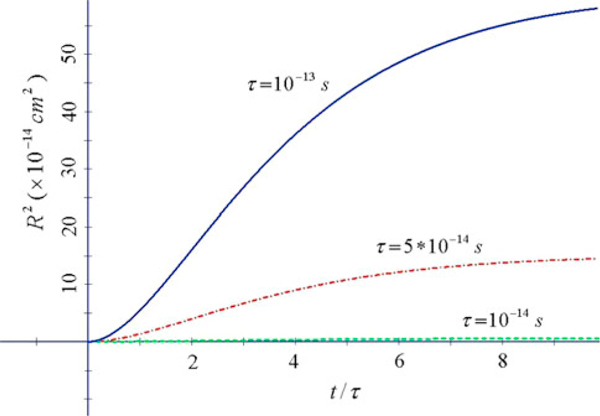
***R*^2^ vs. *x* = *t*/*τ* at constant *ω*_0_*τ*, for 3 values of *τ* (*ω*_0_ = 0.5 × 10^13^Hz *solid*; *ω*_0_ = 10^13^Hz *dot-dashed*; *ω*_0_ = 0.5 × 10^14^Hz *dashed*) for TiO_2_ (*m* = 6*m*_e_, *T* = 300 K)**.

**Figure 4:**
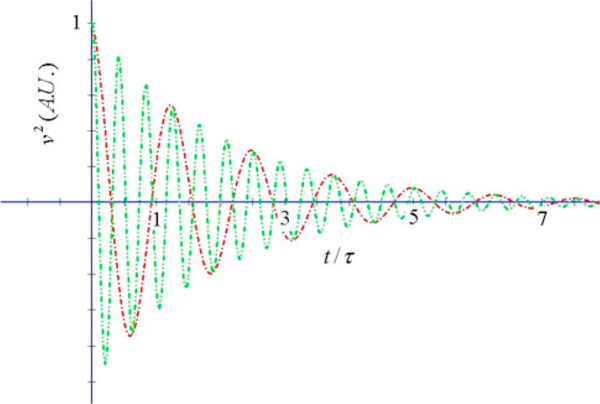
**Velocity correlation function vs. *x* = *t*/*τ* for two values of *α*_R_ ($\alpha_{R}^{2}=4\tau^{2}\omega_{0}^{2}-1$) (*m* = 6*m*_e_, *T* = 300 K)**. Clear exponentially damped oscillations are displayed in this case.

**Figure 5:**
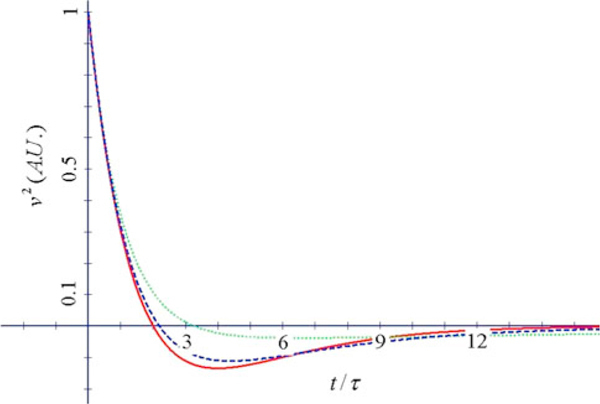
**Velocity correlation function vs. *x* = *t*/*τ* for some values of *α*_I_ ($\alpha_{I}^{2}=1-4\tau^{2}\omega_{0}^{2}$) (*m* = 6*m*_e_, *T* = 300 K)**.

In Figure 1, we show *R*^2^ for doped Si. For this semiconductor, the conductivity is the contribution of two terms, a Drude–Lorentz term and a Drude term [7]. At large times the Drude–Lorentz term leads to an *R*^2^ approaching a constant value (see Figure 2), while the Drude term alone (Figure 1) is the dominant term at large times. Therefore for sufficiently large times, only the Drude term survives.

We observe that the linear relation at large times becomes quadratic at smaller times. The cross-over between the two regimes occurs at times comparable to the scattering time. This means that diffusion occurs after sufficient time has elapsed so that scattering events become significant, while at smaller times the motion is essentially ballistic.

In Figures 2 and 3*R*^2^ saturates at high *t.* The plateau value may assume high values so that *R* may be larger than the size of the nanoparticles composing the films. In general, these features indicate quite enhanced mobility of carriers in the nanoporous films at small times, in contrast with a commonly expected low mobility in a disordered network.

From these figures we can evaluate the diffusion coefficient $D=\frac{1}{2}\frac{\text{d}R^{2}}{\text{d}t}$. It is remarkable that high *D* are obtained at *t*/*τ* of order unity. As an example, from Figure 3 the deduced that diffusion coefficient is *D* ~ 1 cm^2^/s for *τ* = 10^-13^*s*, i.e. comparable to the value ~1 cm^2^/s of the single crystal rutile [2].

From the other hand, much smaller *D* can develop at long times, with values *D* = 10^-4^–10^-6^ cm^2^/s typical of a disordered strong scattering system. So, our results indicate quite different behaviour in Si where normal diffusion occurs, and in TiO_2_ where the Drude–Lorentz model indicates anomalous diffusion.

The physical reason and mechanism of such increase can be traced back to ballistic-like motion of the carriers at early time when scattering is moderate yielding normal diffusion satisfying Eintein's rule and to strong localization due to the scattering at long times with anomalous diffusion with depression of *D*.

Figures 4 and 5 report the behaviour of the velocity correlation function.

We observe that, according to the equations of our model [8,9], the correlation function of velocities is never a single decreasing exponential of time, but it is in general a more complicated combination of exponentials, or an oscillating function of time.

When $1-4\tau^{2}\omega_{0}^{2}>0$, there is a change of sign of velocity with respect to initial velocity, a backscattering mechanism as indicated by Smith [10]: there are two regimes in the temporal response characterized by two different characteristic times, the inverted region being dominated by the longer decay time, and the positive velocity region being due to the shorter time; this region becomes the normal state diffusion region when *ω*_0_ = 0. These two regimes will give rise to small and large diffusion constants respectively, which will be discussed in connection with time-resolved techniques.

When $4\tau^{2}\omega_{0}^{2}-1>0$, we observe the presence of damped oscillations of the velocity in time with strong coupling leading to oscillating currents which average to zero in a sufficiently long interval along with the diffusion constant. This regime appearing at large frequencies for a given time constant does not seem to have been observed in real systems.

The results above give a precise indication on response times of a system subjected to charge motion. In the case of doped Si [7] we have verified the Einstein rule, giving rise to normal diffusion. In the case of TiO_2 _[2,3], anomalous diffusion is found with time-dependent diffusion coefficient vanishing at long times and oscillating behaviour in time of the transport parameters. We have compared our effective diffusion coefficients directly with experimental results [11-13] and with Monte Carlo simulations [14,15], which take into account the overall mechanisms of scattering, including phonons, imperfections and doping centres, and traps, finding that the diffusion coefficient reproduces the values of experimental or simulated coefficients.

We suggest the possibility that our results can give an explanation of the ultra-short times and of high mobilities with which the charges spread in mesoporous nanoparticle TiO_2_ systems, of deep interest in photocatalitic and photovoltaic systems [16,17]. In particular, the relative short times (few *τ*) with which charges can reach much larger distances than typical dimensions of nanoparticles indicate easy diffusion for charges photoproduced inside the nanoparticles towards the surface. The unexplained fact found experimentally of ultrashort injection of charge carriers (particularly in Graetzel's cells) can be related to this phenomenon [16,17].

Similar high diffusivity is found in a number of other devices, i.e. GaAs nanowires and ZnO nanoparticles on which terahertz time-resolved spectroscopy has revealed different time transport regimes with high diffusion processes at short times of the order of the scattering time and longer time localized motion due to the effects of scattering [18,19]. Interpretations of these results in terms of the model suggested here can be given.

Recently, an approach for converting nanoscale mechanical energy into electrical energy has been suggested by using piezoelectric zinc oxide (ZnO) nanowires and TiO_2 _[20]. Such devices have been shown to convert mechanical energy into electric energy with typical ∼1 nW output power per cm^2^ area. These unexpected efficiencies can be explained by anomalous high diffusion in the oxides of the type presented here.

In summary, we have evaluated the correlation functions for systems for which the Drude–Lorentz model is valid through the formulation of a new Drude–Lorentz-like model [8,9], in which such functions can be obtained as complete Fourier transform of the real part of the frequency-dependent complex conductivity *σ*(*ω*). From our results we deduce some important consequences connected with the nanometric film systems, in particular the possibility of a fast response of the transport of charge carriers with a direct consequence for the efficiencies of present devices based on such systems. Of particular interest for nanostructures is the fact that the limiting value of $\langle {\left[\vec{R}\left(t\right)-\vec{R}\left(0\right)\right]}^{2}\rangle$ reaches several nanometers in only few *τ* times, which means that *R* becomes comparable to dimensions of nanoparticles in few scattering events. This implies the possibility of having high mobility of carriers from and towards the surface of nanostructures. This result has possible and interesting implications in photocatalysis and in energy generators, i.e. in photochemical, photoelectrochemical cells and dye-sensitized solar cells (DSSCs) [3]. The principal consequence is the possibility to have high charge conversion efficiencies in particular time intervals. We can thus explain the rather unwaited experimental result that some film oxides as TiO_2_, in which the percolative layer structure would be expected to provide a low mobility, are in reality endowed with high response times of charge injection and with high mobility.
